# Bracing for impact: how shifting precipitation extremes may influence physical climate risks in an uncertain future

**DOI:** 10.1038/s41598-024-65618-9

**Published:** 2024-07-29

**Authors:** Saiful Haque Rahat, Shah Saki, Ummul Khaira, Nishan Kumar Biswas, Ishrat Jahan Dollan, Asphota Wasti, Yuki Miura, Md Abul Ehsan Bhuiyan, Patrick Ray

**Affiliations:** 1 Sustainability, Energy and Climate Change, WSP, New York, NY 10119 USA; 2 Department of Civil and Environmental Engineering, University of Connecticut, 261 Glenbrook Road Unit, 3037, Storrs, CT 06269-3037 USA; 3 National Aeronautics and Space Administration (NASA) Goddard Space Flight Center, Greenbelt, MD USA; 4 Stony Brook University, Stony Brook, NY 10018 USA; 5 HDR Engineering, Sacramento, CA 95833 USA; 6 Mechanical and Aerospace Engineering, Center for Urban Science + Progress, New York University, New York, NY 10012 USA; 7 Climate Prediction Center, National Oceanic and Atmospheric Administration (NOAA), College Park, MD 20742 USA; 8 Department of Chemical and Environmental Engineering, University of Cincinnati, Cincinnati, OH 45221-0012 USA

**Keywords:** Climate sciences, Environmental social sciences, Hydrology, Natural hazards

## Abstract

As extreme precipitation intensifies under climate change, traditional risk models based on the ‘100-year return period’ concept are becoming inadequate in assessing real-world risks. In response, this nationwide study explores shifting extremes under non-stationary warming using high-resolution data across the contiguous United States. Results reveal pronounced variability in 100-year return levels, with Coastal and Southern regions displaying the highest baseline projections, and future spikes are anticipated in the Northeast, Ohio Valley, Northwest, and California. Exposure analysis indicates approximately 53 million residents currently reside in high-risk zones, potentially almost doubling and tripling under 2 °C and 4 °C warming. Drought frequency also rises, with over 37% of major farmland vulnerable to multi-year droughts, raising agricultural risks. Record 2023 sea surface temperature anomalies suggest an impending extreme El Niño event, demonstrating the need to account for natural climate variability. The insights gained aim to inform decision-makers in shaping adaptation strategies and enhancing the resilience of communities in response to evolving extremes.

## Introduction

There remains an unpriced cost associated with climate risk that is steadily accumulating, especially when employing probabilistic methods since past climate patterns alone may no longer reliably indicate future events^1^. Recent catastrophic floods worldwide, including those in Libya, as well as flood events in China, Brazil, and Greece, have highlighted the crucial role of intense heavy rainfall in these disasters^2^. Often, the lack of preparedness for what is traditionally known as a '100-year return period' exacerbates their impact because infrastructure designs assume the probability of such events remains consistent over time^3,4^. However, this stationary assumption crumbles in the context of changing climate conditions, where extreme precipitation events are becoming more frequent and intense. The term ‘100-year return period’ implies a 1% probability event based on limited historical data- an event that may occur more often in the future than once every century^5,6^. Regulatory agencies in both the United States (U.S.)^7^ and the United Kingdom have recognized the non-stationary nature of hydroclimatic conditions under changing climate^8,9^. However, there is a pressing need for risk analysis frameworks that are more adept at handling the escalating intensity of extremes.

Climate risks can be classified into two categories. First, physical climate risks involve potential impacts from climate-related events like flooding, severe storms, or wildfires. These risks are a function of both the likelihood of occurrence and the severity of consequences, which may include asset damage, business disruption, and supply chain interruptions^10,11^. Simultaneously, transition risks are associated with the policy measures implemented to steer the economy away from reliance on fossil fuels and adjust towards a lower-carbon economy^12,13^. As financial institutions strive to align themselves as per the 28th United Nations Climate Change Conference of the Parties (COP28) agreement, they face a critical challenge^14,15^. Relying solely on preparedness strategies based on event-based likelihoods from past data may prove inadequate under changing climate. This predicament presents a paradox that can manifest in two opposing ways. On the one hand, there is the risk of underpredicting climate scenarios, thereby failing to account for the potential magnitude of a future event. Conversely, there is also the danger of overpredicting outcomes, which can result in wasteful investments based on exaggerated risk assessments^16,17^.

For extreme precipitation in particular, climate change has various impacts, which are primarily driven by the increase in air temperature. As the air temperature rises, the water-holding capacity of the atmosphere increases, specifically over the oceans^18,19^. The Clausius–Clapeyron (CC) equation postulates that each 1 °C increase in temperature enables the atmosphere to retain approximately 7% more moisture. In a scenario where the global temperature surpasses the pre-industrial era by 4 °C, the atmospheric water vapor content is estimated to increase by approximately 28%^20,21^. Consequently, this increase can lead to more frequent and intense precipitation, and it is essential to consider climate interactions and local geography to address these changes^22–25^. For instance, low-lying areas like the Gulf Coast, including Houston, become more susceptible to flooding due to its low elevation and high annual rainfall, while California faces the dual challenge of floods and droughts due to elevated temperatures, variable precipitation patterns, and increased evapotranspiration, contributing to the risk of wildfires and ecological disruptions^26–28^.

Climate models employed by scientists generally reach a consensus on the pattern of warming that different regions of the planet will experience; however, the predicted changes in precipitation intensity, timing, and location are considerably less certain^29,30^. The existing models have limited ability to accurately predict extreme precipitation due to insufficient consideration of local hydrological effects^31,32^ and small-scale weather processes^33–35^. Another major challenge lies in the limited availability of reliable observational data and effectively integrating them into the modeling framework. These challenges emerge from issues including insufficient data resolution, variable quality, and lack of consistency over time, making it difficult to incorporate them seamlessly^30,31^.

This study aims to identify these challenges by providing localized insights into the management of physical climate risks across the contiguous United States (CONUS). Using high-resolution (~ 4 km, 1/24th degree) Gridded Surface Meteorological (gridMET) precipitation^36^ data for 1979–2022, it addresses three key questions: (1) How might precipitation extremes shift with changing climate, and what demographic and socio-economic consequences are implicated? (2) What are the short- and long-term impacts on major crops, especially amid severe drought? (3) Considering recent climate events, what is the potential influence of changes in sea surface temperatures on future precipitation and drought extremes? Exploring these questions may provide valuable insights into the non-stationary behavior of precipitation extremes, associated regional vulnerabilities, and sectoral disruptions. Ultimately, these findings can inform the development of adaptive strategies to manage escalating physical climate risks stemming from shifting extremes^37,38^.

## Results

### Changes in regional extreme precipitation patterns

**Figure 1:**
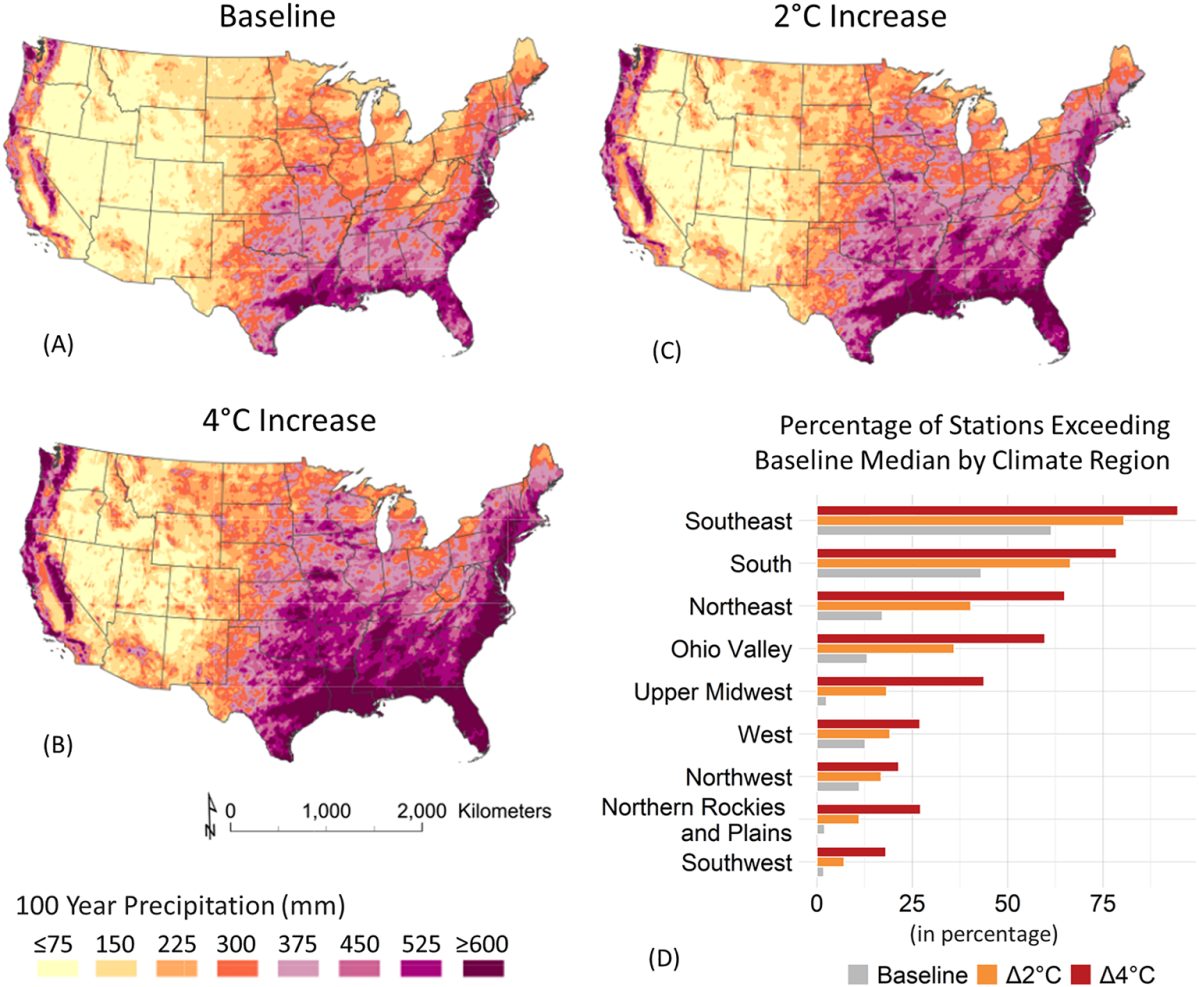
Spatial distribution of 100-year return level for daily precipitation extremes across CONUS. (**A**) Depicts the baseline scenario with a stationary future climate pattern. Panels (**B**) and (**C**) showcase scenarios with increased temperatures (2 °C to 4 °C), incorporating Clausius Clapeyron (CC) scaling through quantile mapping. Panel (**D**) illustrates the percentage of stations exceeding the baseline median by climate region in the CONUS. Among the dataset's 483,879 gridded stations, predominant patterns of extreme precipitation are evident, particularly in the South and Southeastern regions, where approximately 62% and 43% of spatially gridded stations surpass the baseline median of 300 mm for 100-year precipitation extremes. These results underscore the intensification of extreme precipitation events in these regions under increasing temperatures.

Figure 1 illustrates the spatial distribution of the 100-year return level for daily precipitation extremes across different climate regions of the United States, considering non-stationary climatic scenarios where air temperature within the land surface changes from 0 to 4 °C. While the use of the conventional "100-year return period" terminology warrants acknowledgment of its limitations, as discussed in the Introduction section, this standard metric is still prevalent among practitioners when quantifying rare events. Therefore, we utilize this common term to enable comparison, while emphasizing that return levels cannot be treated as fixed under non-stationarity. The data utilized for this analysis comprises 483,879 stations obtained from the gridMET dataset. The baseline scenario adheres to standard techniques presuming stationarity. The increased temperature scenarios account for potential climate change impacts using quantile mapping^39^ (as described in Precipitation Scaling within the Method section), which suggests atmospheric water-holding capacity may increase approximately 7% per 1 °C rise, consequently affecting precipitation^20^.

Under baseline climate conditions (Fig. 1A), projected 100-year precipitation extremes across the CONUS exhibit substantial spatial variability, ranging approximately from 75 to 600 mm. The highest extremes are concentrated in the South and Southeast, with 62% and 43% of gridded stations exceeding the median 300 mm threshold (Fig. 1D). The high extremes in these regions may be influenced by moisture transport from the Gulf of Mexico, which provides a significant moisture source, and the prevalence of intense weather systems like hurricanes and thunderstorms, which bring heavy rainfall and flooding^40,41^. Additionally, regional climate patterns and atmospheric circulation, including factors such as low wind shear and high humidity, may further contribute to the development and intensification of extreme precipitation events in the South and Southeast^42^.

However, even regions like the Northeast, currently experiencing comparatively less intense extremes (17% over median), could see dramatic escalations to 40% and 60% above median with 2 °C and 4 °C warming. These escalations might be propelled by factors such as heightened atmospheric moisture, changes in weather patterns, and the proximity to oceanic moisture sources^43,44^. This intensification pattern extends into the Eastern South region encompassing states such as Arkansas, Oklahoma, and Missouri areas prone to hurricanes and tornadoes possibly due to factors including low wind shear and humidity^45^. These extreme weather phenomena are strongly associated with intense rainfall and elevated flooding, snowstorms, and general water disaster risks^40,46^.

Coastal zones in the Northwest and California also exhibit marked projected spikes due to warming, with percentages rising from 11–13 to 17–27% over baseline medians. The drivers for these increases include the enhanced atmospheric river events, where narrow corridors of concentrated moisture transport lead to extreme precipitation and associated risks like flooding and landslides^47,48^. Given the consistency between the findings and potential future scenarios, it is plausible that the intensity of these events will continue to escalate.

In contrast, regions like the Northeastern Rockies and Plains, Southwestern, and Upper Midwest exhibit relatively muted extremes under baseline climate (Fig. 1A) but are projected to undergo marked increases under elevated warming (Fig. 1B,C, and D). The Ohio Valley, initially with 13% of stations surpassing the median, could see increases to 18% and 44% with 2 °C and 4 °C rises, possibly driven by changes in storm tracks, increased convective activity, and greater moisture availability^49–51^.These results emphasize the need for region-specific strategies to bolster flood resilience and emergency preparedness in response to changing climate conditions. Detailed statistics can be found in Table S-1 within the Supplementary Information Section S-1.

### Population exposure to precipitation extremes under changing climate

**Figure 2:**
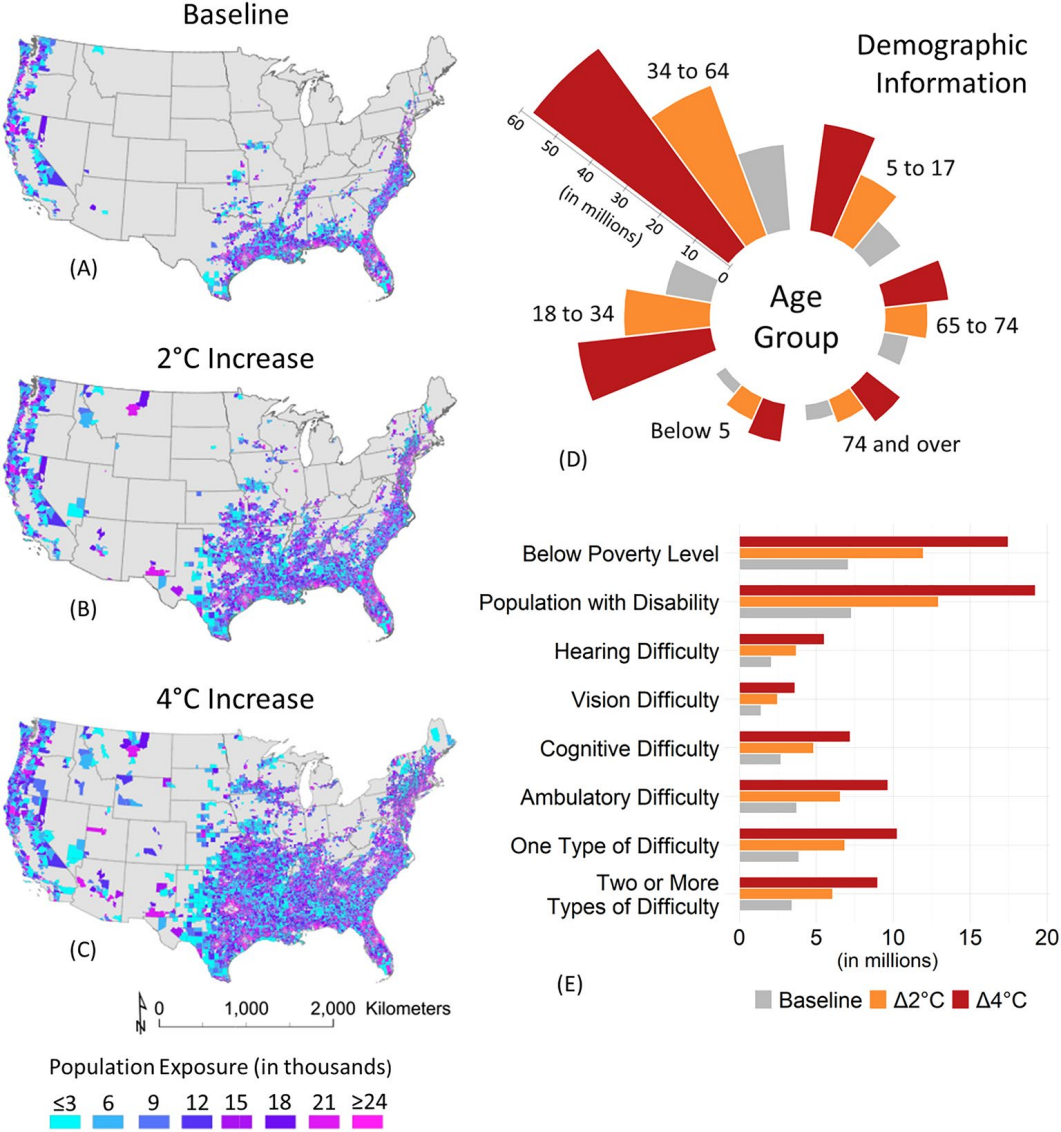
Demographic vulnerability to 100-year precipitation extremes. Panels (**A**)–(**C**) depict population exposure to heightened risks associated with 100-year precipitation extremes under varying temperature scenarios. In the baseline scenario, around 53 million individuals reside in high-risk areas, expected to double to 95 million with a 2 °C temperature increase and triple to 146 million under a 4 °C temperature increase. Panel (**D**) explores demographic characteristics by age group, while Panel (**E**) provides an in-depth analysis of demographic data, including socio-economic status and disability. Current projections indicate that approximately 7 million individuals living below the poverty threshold are exposed to extreme precipitation events. Similar trends are noticeable in different groups of the disabled population as well.

While the previous section highlighted regions susceptible to intensified precipitation extremes, Fig. 2 offers additional insights by quantifying the exposed populations and demographic characteristics at risk. High-risk areas, defined as those surpassing median 100-year return levels based on the census tracts^52^ reveal that approximately 53 million (53,302,365) residents currently reside in these zones (Fig. 2A). With 2 °C warming, the exposure is projected to nearly double, reaching approximately 95 million (94,990,640) and it could almost triple, reaching around 146 million (145,604,690) at 4 °C warming (Fig. 2B and C). The impact of extreme precipitation events is broad, affecting diverse socio-economic groups, including vulnerable populations. Although the majority falls in the age range of 34–64 (Fig. 2D), it is important to note that vulnerable groups such as people with disabilities, children, and older adults may experience disproportionate effects.

A closer examination of the demographic data in Fig. 2E reveals exacerbated risks for marginalized populations under intensified precipitation extremes. Under current projections, approximately 7 million individuals living below the poverty threshold face exposure to extreme precipitation events (Fig. 2E). A 2 °C temperature increase could escalate this number to 12 million, indicating a substantial rise in risk for economically disadvantaged groups. Similarly, exposure for disabled individuals may surge from the current level of 7.5 million to over 13 million with 2 °C of warming. Of the disabled cohort, around 4 million have auditory, visual, cognitive, or ambulatory functional limitations, while 3.5 million experience multiple impairment types (Fig. 2E). Vulnerable populations, particularly the elderly and disabled, often experience access barriers and disruptions in care services during extreme weather occurrences. Furthermore, urban populations of lower socioeconomic status frequently contend with crowded emergency shelters and adverse health outcomes, whereas middle-income households may incur property damage but possess insurance to aid recovery. The results suggest a growing number of vulnerable people may become susceptible to these risks as precipitation extremes intensify under climate change. Further detailed statistics can be found in Table S-2A and S-2B within the Supplementary Information Section S-2.

### Socioeconomic perspective under precipitation extremes in major U.S. cities

**Figure 3:**
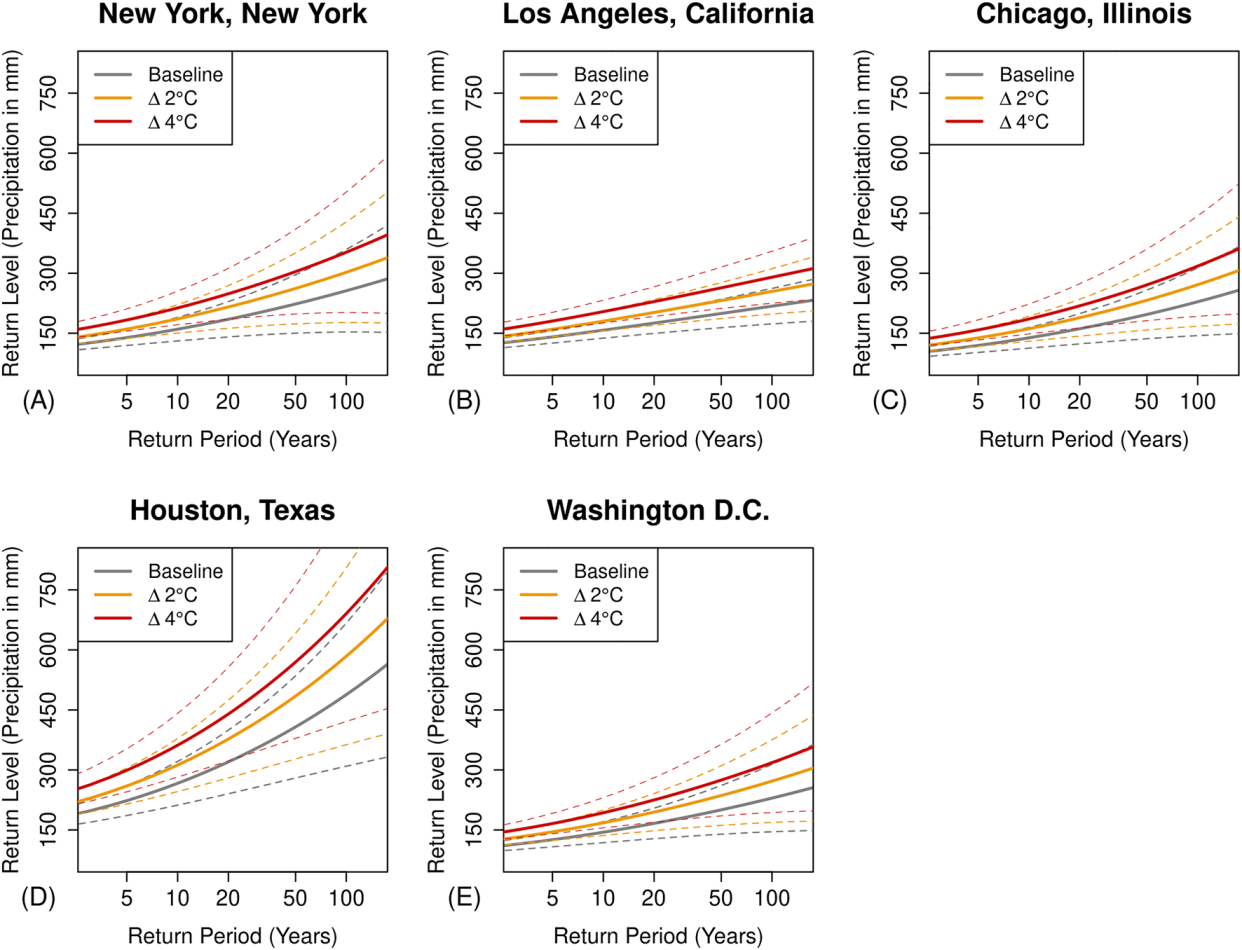
Changes in daily extreme precipitation events under warming scenarios averaged across gridded stations for major cities: (**A**) New York, New York; (**B**) Los Angeles, California; (**C**) Chicago, Illinois; (**D**) Houston, Texas; (**E**) Washington, D.C. Return periods with 95% confidence intervals are indicated as dotted gray lines. Increasing temperatures intensify the magnitude of 100-year extreme precipitation events, projecting approximately a 33% increase for New York City and a 35% increase for Houston in a changing climate. Comparatively less pronounced increases are observed for Los Angeles (~ 12%), Chicago (~ 25%), and Washington D.C. (~ 15%).

Building on the nationwide demographic analysis, we take a finer-grained socioeconomic perspective of exposure disparities across major US cities (i.e., four cities ranked by population size including the national capital). Figure 3 illustrates the variations in daily extreme precipitation events averaged across the gridded stations for major cities under increasing temperature scenarios. Implementing quantile mapping^39^, on high-resolution gridded data, the projections reveal precipitation extremes amplifying by approximately 33% (Fig. 3A) in New York City (from 280 to 380 mm) and 35% (Fig. 3D) in Houston (from 480 to 650 mm) for 4 °C warming compared to the baseline scenario. While the extent of the increase in extreme precipitation is relatively less pronounced for cities like Los Angeles (~ 12%), Chicago (~ 25%), and Washington D.C. (~ 15%), the findings still indicate that existing infrastructure design guidelines (Fig. 3B,C and D), based on 100-year events from observed data in the baseline scenarios, may prove inadequate in withstanding projected extreme precipitation events in a changing climate scenario.

**Figure 4:**
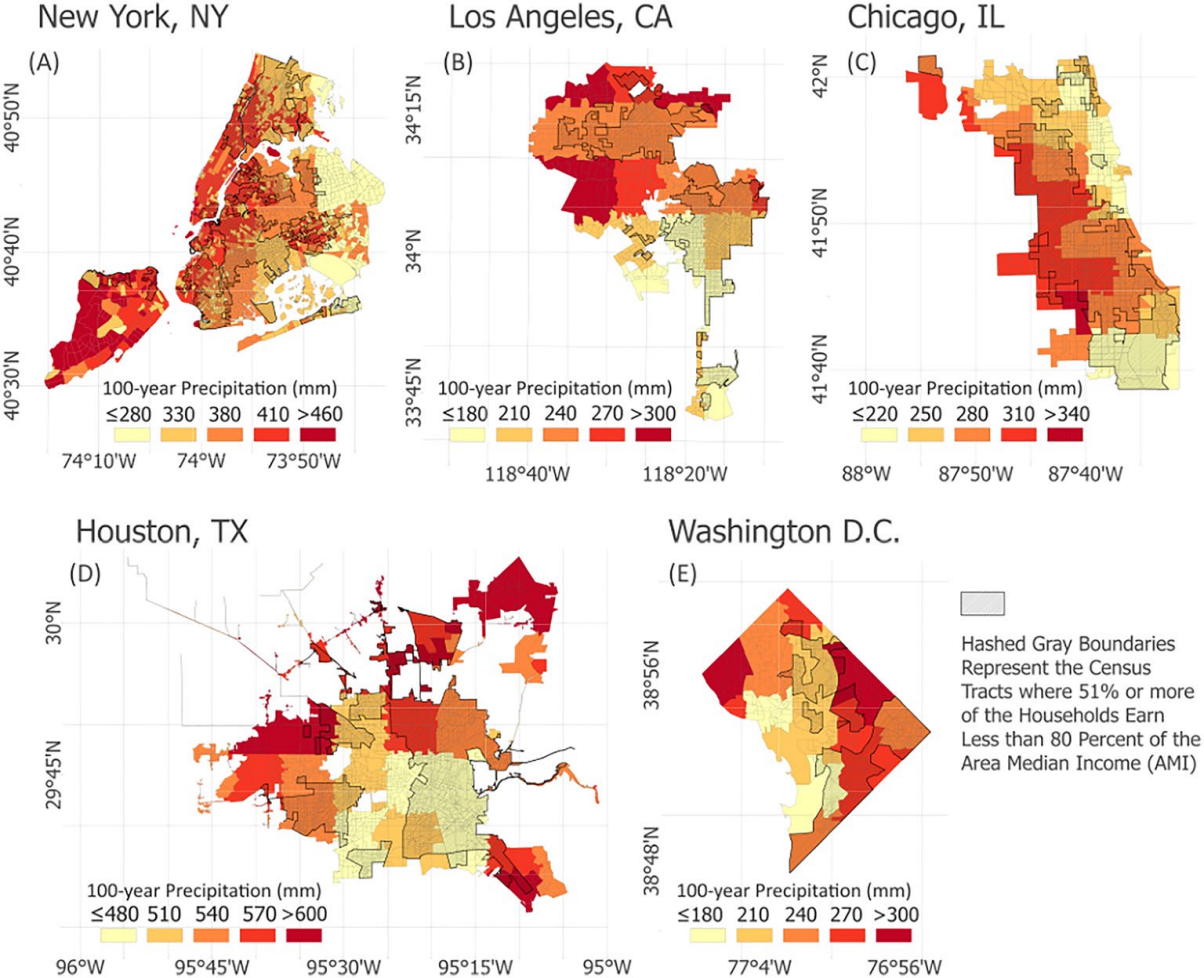
Geospatial analysis of socioeconomic disparities in exposure to extreme precipitation in major U.S. Cities. (**A**) New York, New York; (**B**) Los Angeles, California; (**C**) Chicago, Illinois; (**D**) Houston, Texas; (**E**) Washington, D.C. Hashed gray boundaries indicate census tracts with 51% or more of households earning less than 80% of the Area Median Income (AMI). The findings reveal vulnerabilities across various income levels within urban areas, with approximately 37% of below-AMI households in New York, 21% in Los Angeles, 22% in Chicago, 35% in Houston, and 42% in Washington D.C. exposed to precipitation levels exceeding their respective baseline 100-year medians.

Fine-grained geospatial analysis in Fig. 4 reveals substantial socioeconomic disparities in exposure to extreme precipitation intensification across major US cities. This assessment is performed utilizing census tracts and considering a non-stationary scenario characterized by a temperature increase of 0.8 °C from 1986 to 2016 as found in the literature^53^. Hashed gray boundaries represent the Census tracts where 51% or more of the Households earn less than 80 percent of the Area Median Income (AMI)^54^. The results shed light on the vulnerabilities experienced related to extreme precipitation by diverse household income communities within these urban centers.

For instance, in the case of New York City (Fig. 4A), 37% of low-income households are situated in areas that exceed the 100-year baseline for extreme precipitation (specifically, a threshold of 280 mm, as depicted in Fig. 3A). A comparable situation is observed in Los Angeles (Fig. 4B), where 22% of low-income households reside in regions that surpass the 180 mm baseline (Fig. 3B). Similarly, larger fractions of lower-income households in Houston (31% vs. 35%), Chicago (40% vs. 9%), and Washington D.C. (42% vs. 38%) are situated in locations expecting intensified extremes beyond 100-year thresholds. Poorer neighborhoods often lack resources to manage flooding and stormwater, facing decaying infrastructure, increased property damage, and threats to human health. In contrast, higher-income regions may absorb impacts with greater financial capacity but exposure to such intensified precipitation extremes may lead to potential declines in property valuations and increases in insurance costs^55^. These results highlight climate change as a catalyst for worsening pre-existing inequalities, underscoring the pressing need to prioritize infrastructure improvements, community resilience programs, and policy interventions in vulnerable lower-income areas^55,56^.

### Impacts of severe drought events on agriculture

**Figure 5:**
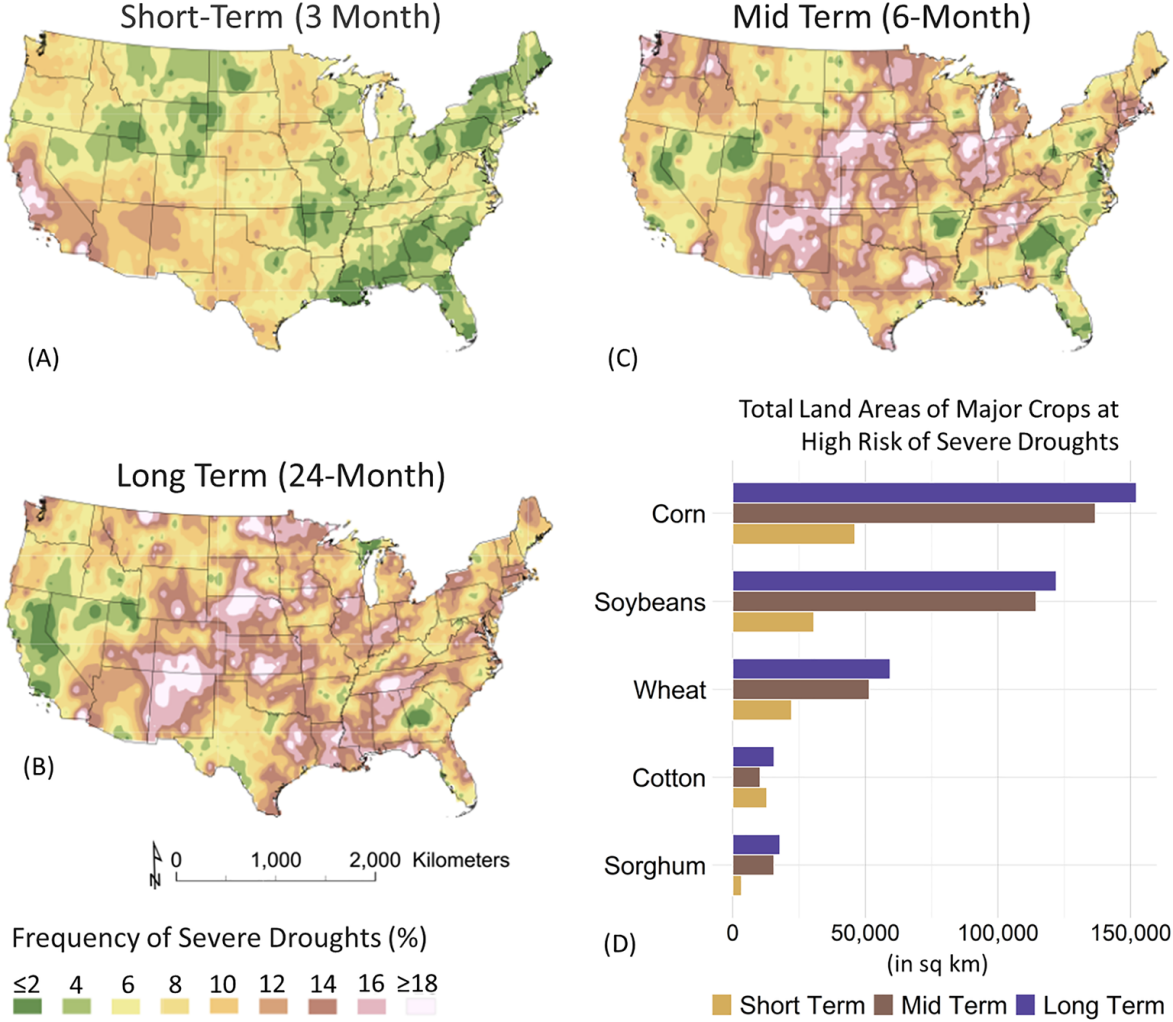
Frequency of severe drought events in the United States under non-stationary climate scenarios. Panels (**A**)–(**C**) illustrate short-term, mid-term, and long-term drought events spanning 3, 6, and 24 months, respectively. Severe drought event frequency is represented as a percentage, assessed using the Standardized Precipitation Index (SPI) with a classification threshold below -1.5. The results emphasize elevated rates of long-term severe droughts (> 18%) in both the southwestern region (encompassing New Mexico and Colorado) and the midwestern region (comprising Kansas, Missouri, Iowa, and Nebraska). In Panel (**D**), the total land areas of major crops in the United States vulnerable to high-risk severe droughts are presented. Notably, approximately 37% (367,184 sq-km) of the considered agricultural land is susceptible to long-term severe drought, while around 12% (115,276 sq-km) faces the risk of short-term drought events.

While the previous sections concentrated on the extremes of amplified precipitation, climate change also exacerbates the opposing extremes—intensifying droughts. Figure 5 depicts an analysis of severe drought occurrences over various time scales, including short-term (3 months in Fig. 5A), mid-term (6 months in Fig. 5B), and long-term (24 months in Fig. 5C) periods. To identify severe drought events, the study utilized the Standard Precipitation Index (SPI), categorizing events with an SPI value below − 1.5 as severe droughts^57^. This classification accounts for a documented temperature increase of 0.8 °C between 1986 and 2016, drawing from historical observations within the United States^53^. Analysis of US drought events reveals intensified risks in localized hotspots, driven by regional climate factors. The semi-arid Southwest exhibits increased vulnerability to prolonged 24-month droughts (Fig. 5C) with over 18% occurrences^58,59^, while the Midwest, particularly in its western regions, experiences heightened long-term drought frequency possibly due to exposure to severe weather patterns^58,59^. Conversely, the Midwest, particularly its western areas, faces drought events due to its proximity to the Great Plains, an area prone to severe weather patterns including droughts^51^. However, California faces a higher short-term drought frequency (Fig. 5A), likely from highly variable precipitation patterns^60^.

Overlaying these high-resolution drought patterns onto cropland data from the United States Department of Agriculture (USDA) reveals significant risks to major crops (corn, soybeans, wheat, cotton, and sorghum) by production value^61^. Approximately 37% (around 367,000 sq km) of the studied cropland is susceptible to long-term droughts, while 12% (approximately 115,000 sq km) is vulnerable to short-term drought events. This may pose a threat to agricultural yield, potentially impacting regional food security and influencing grocery prices^62^. Adaptive practices tailored to vulnerable regions are essential to secure the nation's agricultural productivity against drought impacts. For further details, please refer to Table S-3 within the Supplementary Information Section S-3.

### Influence of sea surface temperature on precipitation extremes and droughts

**Figure 6:**
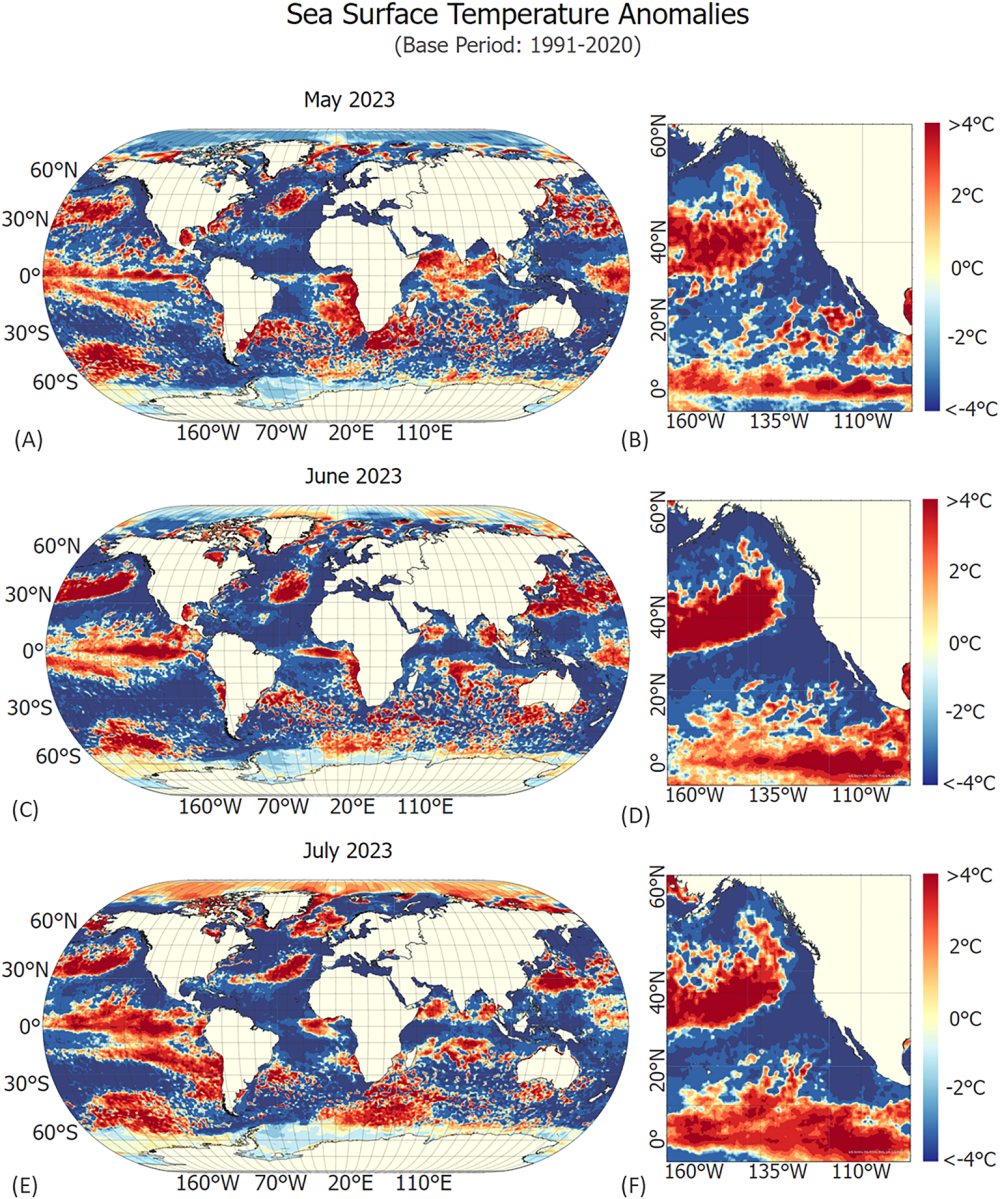
Sea Surface Temperature (SST) Anomaly Data for May, June, and July 2023 relative to the Base Period of 1991 to 2020. Panel (**A**) presents anomalies for the month of May on a global scale, while Panel (**B**) offers a detailed view of the equatorial Pacific Ocean near the Pacific Coast. Similarly, Panels (**C**) and (**D**) depict SST anomalies for June, and Panels (**E**) and (**F**) show the same for July. Positive anomalies (+ 3 to >  + 4  °C) observed in the central and eastern equatorial Pacific Ocean near the equator are indicative of El Niño events, known for their far-reaching impacts on global temperature and precipitation patterns. These findings align with reports from the WMO and NOAA, indicating a probable increase in global SST since April 2023, reaching a record high in July.

The influence of larger-scale climatic phenomena, such as the El Niño–Southern Oscillation (ENSO), on future extreme precipitation and drought events warrants consideration^63^. An analysis of Sea Surface Temperature (SST) anomalies in the central and eastern equatorial Pacific for May (Fig. 6A and B), June (Fig. 6C and D), and July 2023 (Fig. 6E and F) reveals positive anomalies ranging from + 3 to greater than + 4 °C compared to the 1991–2020 baseline period. These findings align with reports from the World Meteorological Organization (WMO) and National Oceanic and Atmospheric Administration (NOAA) showing a persistent rise in global SST since April 2023, peaking in July at a new record high^64,65^.

Such anomalies are associated with El Niño events that have widespread impacts on global temperature and precipitation patterns, consequently affecting socioeconomic and environmental systems. Historical records reveal that strong El Niño events, such as those in 1982–1983 and 1997–1998, led to heavy flooding along the western coast of South America, while regions like Australia and Indonesia suffered from severe droughts^66,67^.

Within the United States, the influence of El Niño is relatively weak during the summer months but becomes more pronounced from late fall through spring. Moderate to strong El Niño conditions in the fall and winter are typically associated with above-average precipitation in the Gulf Coast region and southern California, while below-average precipitation is observed in the Pacific Northwest and Ohio Valley. Furthermore, the likelihood of warmer-than-average temperatures across the northern United States increases during El Niño winters^67^. In contrast to El Niño years, La Niña years (with negative SST anomalies) and ENSO neutral years (with SST anomalies close to 0) also have the potential to influence the extreme precipitation patterns^66^. The record high sea surface temperatures (SSTs) seen in 2023 suggest that an extreme El Niño event, one of the most intense on record, may soon occur. This highlights the importance of considering natural climate variability, such as the ENSO, in short-term and long-term risk management strategies. Accounting for these phenomena may facilitate better anticipation of location-specific precipitation extremes and associated economic impacts.

## Discussion

As we witness the persistent warming trend over the past decade, the UN Secretary-General has recently declared a transition from the "Global Warming" era to what is now referred to as the "Global Boiling" era^68^. The implications of this transition on the frequency and intensity of future extreme precipitation events continue to be a pressing concern. Despite prior efforts^24,25, 69^ there is limited understanding of physical climate risk into relevant practices for the factors related to local climatic processes. By utilizing high-resolution data and adopting a non-stationary approach, this study offers valuable insights into the physical climate risks linked to extreme precipitation events at a localized level.

Specifically, addressing the research questions, the study reveals pronounced regional variability in precipitation extremes, identifying coastal and Southern hotspots where infrastructure enhancement should be prioritized locally. The exposure quantification indicates over 50 million US residents currently face high risks that may double with 2 °C warming, revealing socioeconomic vulnerabilities requiring targeted policy interventions^70^. Additionally, analysis of cropland exposure reveals over a third of major crop areas are susceptible are vulnerable to heightened multi-year droughts, raising concerns about declines in crop yields, and food security^51^. Detecting an impending extreme El Niño event underscores the interplay between natural variability and anthropogenic forcing. Incorporating such interconnected climatic dynamics is imperative for robust climate risk assessment. The key takeaways indicate escalating extremes requiring adaptive strategies to build resilience. Moving forward, coupling localized projections with sectoral impact models could further quantify risks to assets, operations, supply chains, and communities^71^. As climate risk disclosure expands, findings like these will prove increasingly valuable for balancing infrastructure costs, business risks, and environmental needs under more frequent and intense extremes^27,72^.

While providing valuable insights, this work has limitations warranting acknowledgment. The methodology primarily focuses on atmospheric water-holding capacity, omitting certain climatic factors, feedbacks, regional weather patterns, and micro-hydrological variables that could influence results^73,74^. The drought index relies solely on precipitation without incorporating groundwater, evapotranspiration, and water availability^27,28^. Additionally, non-climatic factors such as land use, environmental policies, and infrastructure development were beyond the scope of this study^75^. Furthremore, it is worth noting that the application of Clausius–Clapeyron scaling in assessing extreme precipitation events might encounter challenges. This is due to its assumption of uniform atmospheric conditions, which may not hold true during events characterized by localized intense convective processes and other influencing factors^76,77^. Recognizing these caveats provides important context around the findings and points toward opportunities to enhance the reliability of climate risk assessment in the future.

Overall, this study advances a localized understanding of intensifying precipitation extremes and associated vulnerabilities. The insights gained directly inform adaptation strategies aimed at strengthening resilience to physical climate risks across sectors. Addressing the identified limitations represents an avenue for additional progress.

## Method

### Precipitation scaling

In this study, quantile mapping is employed to rescale the distribution of precipitation from the historical dataset, aiming to emulate the impact of increased temperatures on precipitation patterns due to a higher moisture-holding capacity of the atmosphere, known as CC scaling. Building upon previous research^20,39^, CC scaling assumes a 7% increase in the water-holding capacity of the atmosphere per degree Celsius of temperature rise and is quantified as a percentage change in the statistical moments and quantiles of the precipitation distribution per degree of temperature rise. By quantifying this relationship, it becomes possible to estimate the potential changes in precipitation under different climate scenarios. To identify extreme precipitation events, the 99.9th percentile of non-zero precipitation values is selected. This threshold is chosen to focus on the most extreme events within the dataset. To fit the data into a suitable probabilistic model, a Generalized Pareto Distribution (GPD) model^78^ is employed, represented by the following equation:

1 $$P;\mu , \sigma , \xi = \left( {1/\sigma } \right) * \left[ {\left( {1 + \xi \left( {\left( {x - \mu } \right)/\sigma } \right)} \right) \wedge \left( { - \left( {1/\xi } \right) - 1} \right)} \right], \;for\; \xi \ne 0$$

where P represents the vector of precipitation values for each data point, μ denotes the location parameter, σ represents the scale parameter, ξ represents the shape parameter.

For our analyses, we utilized a continuous daily precipitation dataset from 1979 to 2022, encompassing 483,879 stations across the CONUS region. We applied quantile mapping techniques separately for each station to assess the impact of temperature increases on extreme precipitation values. Initially, we fitted a GPD model to the time series data for each station and calculated the 1% annual exceedance probability, referred to as the 100-year precipitation event, aligning with traditional terminology. This baseline scenario assumes stationarity in the climate data, with no future changes. Next, we applied quantile mapping to each of the 483,879 fitted GPD to estimate the 100-year precipitation values under temperature increases of 2 and 4 °C. This approach provides insights into how extreme precipitation events might change, considering that the water-holding capacity of the atmosphere increases by approximately 7% per degree Celsius, leading to an estimated increase of 14% and 28% for 2 and 4 °C, respectively.

#### Drought index

To assess regional drought conditions, we incorporate the SPI, which serves as a standardized measure for characterizing meteorological drought on a range of timescales^57^. In this study, we consider four distinct accumulation periods: the short-term periods of 3 months and 6 months, as well as the long-term periods of 12 months and 24 months. These accumulation periods enable us to evaluate drought conditions across various time scales, capturing both immediate and prolonged drought phenomena. The SPI is computed using the equation:

2 $${\text{SPI}} = \left[ {{\text{Z }} - \, \mu } \right]/\sigma$$

where Z represents the accumulated precipitation over the specified accumulation period, μ denotes the long-term mean precipitation for the same accumulation period, and σ signifies the standard deviation of precipitation for the accumulation period under consideration. Based on the calculated SPI values, we identify severe drought events by utilizing a predefined threshold of SPI index values smaller than − 1.5^57^. By establishing such thresholds, we can delineate the severity of drought events and facilitate a robust understanding of regional drought conditions.

### gridMET dataset

gridMET is a comprehensive dataset that offers daily high-spatial resolution (~ 4 km, 1/24th degree) surface meteorological data for the contiguous United States since 1979^36^. It encompasses primary climate variables, including temperature, precipitation, radiation, wind, and humidity, as well as derived variables such as evapotranspiration and drought severity index. The dataset is generated by combining gridded climate data from PRISM with regional reanalysis data, producing a spatially and temporally complete, high-resolution dataset. Extensive validation against weather stations has been performed to ensure accuracy^79^. The high resolution of gridMET makes it particularly well-suited for our study, capturing localized climate variations and extremes. This allows us to analyze spatial heterogeneity in precipitation patterns, the impacts of microclimatic conditions on agricultural productivity, and regional vulnerabilities to climate change with greater precision. Additionally, gridMET's comprehensive coverage and long temporal span provide an invaluable tool for studying climate trends and developing adaptive strategies over multiple decades. However, it should be noted that gridMET may have limitations in capturing microclimates below its native resolution and mesoscale terrain influences. Additionally, solar radiation data provided is for a planar surface, requiring adjustments for topographic effects. Despite these considerations, gridMET serves as a valuable resource for ecological, agricultural, and hydrological modeling in scientific research.

### Census tract dataset

This study employs 2020 United States Census tract data to perform an extensive analysis of population exposure and socio-economic perspectives within the context of extreme precipitation events^52^. Census tracts, designed as geographical units for census purposes, serve as a critical framework for studying localized demographic and socio-economic factors, making them a valuable resource for this research. The data sources, including the 2020 Census's Redistricting Data Summary Files and the Demographic and Housing Characteristics File, are publicly accessible, ensuring transparency and reproducibility. The study's emphasis on the significance of census tracts highlights their role in understanding the diverse impacts of extreme weather events on different communities, contributing to evidence-based policy decisions. The incorporation of the 2020 Census Demographic Data Map Viewer further enhances the study's analytical process, offering an interactive platform for visualizing and interpreting data at various geographical scales, thereby enriching the depth and quality of the analysis.

### NOAA OISST high-resolution dataset

The NOAA Optimum Interpolation Sea Surface Temperature (OISST) dataset offers daily, high-resolution (0.25°) SST data derived from both in situ and satellite observations, covering the global oceans from September 1981 to the present. With a spatial resolution of 0.25° latitude/longitude, it provides more detailed SST information than the standard NOAA OISST data at 1° resolution. The dataset features a daily temporal resolution, generating a new SST map each day using observations from the previous day, facilitating the analysis of daily SST variability. Employing statistical methods, the dataset combines data from various satellite sensors and in situ platforms like ships and buoys, with advanced quality control ensuring a high-resolution blended SST analysis to minimize observational errors and gaps. For this study, the dataset was utilized to extract spatial and temporal information on ocean surface temperatures across global oceans for multiple decades, to comment on potential climatological events (e.g., El-Nino, PDO) that may influence the extreme precipitation globally and regionally within the United States^80^.

### Supplementary Information

Supplementary Information.

## Data Availability

The data utilized in this analysis study are openly accessible and thoroughly described in the method section. Any additional data or analytical code supporting the study's findings, not featured in the published article, can be obtained from the corresponding author upon request.
