# Author Correction: Bracing for impact: how shifting precipitation extremes may influence physical climate risks in an uncertain future

**DOI:** 10.1038/s41598-024-70360-3

**Published:** 2024-08-28

**Authors:** Saiful Haque Rahat, Shah Saki, Ummul Khaira, Nishan Kumar Biswas, Ishrat Jahan Dollan, Asphota Wasti, Yuki Miura, Md Abul Ehsan Bhuiyan, Patrick Ray

**Affiliations:** 1 Sustainability, Energy and Climate Change, WSP, New York, NY 10119 USA; 2 Department of Civil and Environmental Engineering, University of Connecticut, 261 Glenbrook Road Unit, 3037, Storrs, CT 06269‑3037 USA; 3 National Aeronautics and Space Administration (NASA) Goddard Space Flight Center, Greenbelt, MD USA; 4 Stony Brook University, Stony Brook, NY 10018 USA; 5 HDR Engineering, Sacramento, CA 95833 USA; 6 Mechanical and Aerospace Engineering, Center for Urban Science + Progress, New York University, New York, NY 10012 USA; 7 Climate Prediction Center, National Oceanic and Atmospheric Administration (NOAA), College Park, MD 20742 USA; 8 Department of Chemical and Environmental Engineering, University of Cincinnati, Cincinnati, OH 45221‑0012 USA

Correction to: *Scientific Reports* 10.1038/s41598-024-65618-9, published online 29 July 2024

The original version of this Article contained errors in the Figure legend of Figure 2.

The legend of Figure 2:

“Demographic vulnerability to 100-year precipitation extremes. Panels (**A**)–(**C**) depict population exposure to heightened risks associated with 100-year precipitation extremes under varying temperature scenarios. In the baseline scenario, around 25 million individuals reside in high-risk areas, expected to double to 49.5 million with a 2 °C temperature increase and triple to 78.5 million under a 4 °C temperature increase. Panel (**D**) explores demographic characteristics by age groups, while Panel (**E**) provides an in-depth analysis of demographic data, including socio-economic status and disability. Current projections indicate that approximately 7 million individuals living below the poverty threshold are exposed to extreme precipitation events. Similar trends are noticeable in different groups of disabled population as well.”

now reads:

“Demographic vulnerability to 100-year precipitation extremes. Panels (**A**)–(**C**) depict population exposure to heightened risks associated with 100-year precipitation extremes under varying temperature scenarios. In the baseline scenario, around 53 million individuals reside in high-risk areas, expected to double to 95 million with a 2 °C temperature increase and triple to 146 million under a 4 °C temperature increase. Panel (**D**) explores demographic characteristics by age group, while Panel (**E**) provides an in-depth analysis of demographic data, including socio-economic status and disability. Current projections indicate that approximately 7 million individuals living below the poverty threshold are exposed to extreme precipitation events. Similar trends are noticeable in different groups of the disabled population as well.”

The original Article has been corrected.

