# Supplementary material for: Bracing for impact: how shifting precipitation extremes may influence physical climate risks in an uncertain future
Source: Sci Rep. 2024 Jul 29;14:17398. doi: 10.1038/s41598-024-65618-9 (PMC11286852; doi:10.1038/s41598-024-65618-9)
Supplement: Supplementary file 1 — Supplementary Information. [file 41598_2024_65618_MOESM1_ESM.docx]

# S-1 Changes of Precipitation Extremes within Gridded Station Data Across U.S. Climate Regions

Table S-1 presents a comprehensive analysis of the impact of climate change scenarios on gridded station data across various regions in the United States, with a focus on precipitation extremes. The table includes climatic regions, along with the states considered within each region. It provides information on the percentage of gridded stations that exceed the baseline median temperature for three different scenarios: Baseline, a 2-degree Celsius increase (Δ2°C), and a 4-degree Celsius increase (Δ4°C). This table offers valuable insights into how temperature increases in these scenarios influence the percentage of stations that exceed the baseline median for precipitation extremes in different regions.

**Table S-1 Percentage of the Grided Stations Exceeding the Baseline Median of 100-year Precipitation Extremes under Climate Change Scenarios across the United States.**

| Regions | States Considered | Percentage of the Gridded Stations Exceeding Baseline Median (%) | | |
| --- | --- | --- | --- | --- |
|  |  | Baseline | Δ2°C | Δ4°C |
| Northeast | CT, DE, ME, MD, MA, NH, NJ, NY, PA, RI, VT | 17.167 | 40.368 | 65.006 |
| Upper Midwest | IA, MI, MN, WI | 2.509 | 18.278 | 43.827 |
| Ohio Valley | IL, IN, KY, MO, OH, TN, WV | 13.176 | 36.999 | 59.818 |
| Southeast | AL, FL, GA, NC, SC, VA | 61.458 | 80.499 | 94.662 |
| Northern Rockies and Plains | MT, NE, ND, SD, WY | 2.033 | 11.077 | 27.183 |
| South | AR, KS, LA, MS, OK, TX | 43.076 | 66.475 | 78.557 |
| Southwest | AZ, CO, NM, UT | 1.816 | 7.159 | 18.098 |
| Northwest | ID, OR, WA | 11.134 | 16.876 | 21.458 |
| West | CA, NV | 12.672 | 19.158 | 27.034 |

# S-2 Supplemental Information on the Population Exposure Analysis under Precipitation Extremes

Table S-2A displays demographic type distributions within a population under varying temperature and precipitation extremes. Demographic categories include individuals below the poverty level, those with disabilities, and those experiencing hearing, vision, cognitive, and ambulatory difficulties, as well as individuals with one type of difficulty. The table shows the percentage or proportion of these demographic groups under baseline conditions, Δ2°C, and Δ4°C scenarios. This table offers insights into the potential impact of different climate scenarios on demographic categories, influenced by factors such as socioeconomics and climate change.

**Table S-2A: Demographic Vulnerability for Precipitation Extremes Under Variable Climate Scenarios**

| Demographic Type | Baseline  *(in millions)* | Δ2°C  *(in millions)* | Δ4°C  *(in millions)* |
| --- | --- | --- | --- |
| Below Poverty Level | 7.086 | 11.975 | 17.483 |
| Population with Disability | 7.305 | 12.94 | 19.257 |
| Hearing Difficulty | 2.091 | 3.714 | 5.53 |
| Vision Difficulty | 1.422 | 2.49 | 3.613 |
| Cognitive Difficulty | 2.718 | 4.833 | 7.209 |
| Ambulatory Difficulty | 3.737 | 6.567 | 9.674 |
| One Type of Difficulty | 3.877 | 6.865 | 10.265 |
| Two or More Types of Difficulty | 3.428 | 6.075 | 8.992 |

Table S-2B provides a comparative analysis of demographic data within different age groups across three scenarios: the Baseline, Δ2°C, and Δ4°C. The table illustrates the percentages or proportions of individuals within these age groups under the specified temperature conditions. It offers valuable insights into how changes in precipitation extremes, driven by temperature scenarios, can impact the distribution of age groups within a population.

**Table S-2B: Demographic Information by Age Group Under Variable Climate Scenarios**

| Age Group | Baseline  *(in millions)* | 2°C  *(in millions)* | 4°C  *(in millions)* |
| --- | --- | --- | --- |
| Below 17 | 3.144 | 5.586 | 8.565 |
| 5 to 17 | 9.085 | 16.220 | 24.890 |
| 18-34 | 11.134 | 19.807 | 30.671 |
| 35 to 64 | 20.639 | 36.983 | 56.841 |
| 65 to 74 | 5.647 | 9.944 | 14.956 |
| 74 and over | 3.652 | 6.450 | 9.681 |
| Total | 53.302 | 94.990 | 145.604 |

# S-3. Crop Vulnerability Across Different Drought Durations

Table S-3 presents information on "Drought Duration" and the corresponding "Vulnerable Total Area by Crop Type" in square kilometers (sq-km). The table is structured to display data for three different drought duration periods: 24 months, 6 months, and 3 months. For each duration period, the table provides the vulnerability statistics for major crop types, including corn, soybeans, wheat, cotton, and sorghum. The "Total Crop Area" is calculated for each duration period, and the "% Crop Area Vulnerable" indicates the proportion of the total crop area that is vulnerable to drought conditions. This data is valuable for understanding the susceptibility of different crop types to drought events over varying time frames and helps assess the potential impact on agricultural production and food security.

**Table S-3: Vulnerable Total Area by Crop Type for Different Drought Conditions**

| Drought Duration | Vulnerable Total Area by Crop Type (sq-km) | | | | | | |
| --- | --- | --- | --- | --- | --- | --- | --- |
|  | *Corn* | *Soybeans* | *Wheat* | *Cotton* | *Sorghum* | *Total Crop Area* | *% Crop Area Vulnerable* |
| 24 Months | 152,230 | 121,966 | 59,441 | 15,673 | 17,875 | 367,184 | 37.08 |
| 6 Months | 136,713 | 114,358 | 51,483 | 10,395 | 15,601 | 328,550 | 33.20 |
| 3 Months | 46,094 | 30,602 | 22,243 | 12,942 | 3,394 | 115,276 | 11.64 |
